# On the Authority of Advance Euthanasia Directives for People with Severe Dementia: Reflections on a Dutch Case

**DOI:** 10.1002/hast.1418

**Published:** 2022-10-13

**Authors:** Henri Wijsbek, Thomas Nys

**Keywords:** dementia, euthanasia, advance directives, personal identity, response shift, normative authority, bioethics

## Abstract

*In 2016, a Dutch physician complied with the advance euthanasia directive of a patient with severe dementia. In the ensuing lawsuit, the physician was charged principally with termination of life on request, with euthanasia, that is, and alternatively with murder. A district court acquitted her of both charges, a decision later upheld by the Dutch Supreme Court*.

*Euthanasia is still a criminal offense in the Netherlands, but in 2002, a new euthanasia act came into effect allowing a physician to perform euthanasia if she has fulfilled six due‐care criteria, the two most important of which are that she must be satisfied that the patient's request is voluntary and well considered and that the patient's suffering is unbearable, with no prospect of improvement. Neither of these two criteria is controversial; particular cases are controversial when it is disputed that these criteria were fulfilled. Other euthanasia cases, however, are controversial because they belong to a category that is contentious for independent reasons. One such disputed category is euthanasia performed on incompetent patients with an advance directive*.

*Since determining whether these criteria are fulfilled in a particular case depends on the particular circumstances, we describe this 2016 case in detail. We then focus on three general philosophical reasons for doubting the validity of advance euthanasia directives for incompetent patients: the someone else problem, the problem of response shift, and the problem of normative authority. These problems have been discussed before, but our primary aim is to show how they are connected. Taken together, we believe that our two lines of argument make a strong moral case for supporting the Dutch Supreme Court's decision*.

## Article

In 2016, the Dutch geriatrician Marinou Arends terminated the life of a seventy‐four‐year‐old woman with severe dementia, whom we shall call Mrs. Cornel.^1^ Mrs. Cornel had been diagnosed with Alzheimer's four years earlier, though her symptoms dated from 2007. When she got her diagnosis, she drafted an advance euthanasia directive, which she confirmed a year before her death, emphatically requesting euthanasia if she had to be admitted to a nursing home and could no longer stay with her husband, to whom she had been married for over fifty years without their having spent a single night apart. Alzheimer's ran in her family: two of her brothers had died of Alzheimer's, and so had her mother, after having spent her last twelve years in a nursing home. Seeing her mother deteriorate had been a traumatic experience for Mrs. Cornel, and under no circumstances did she want to go through the same process.

The case stirred up a fierce debate among professionals in the Netherlands as well as among the public, primarily because Mrs. Cornel was incompetent and could not confirm her written request when the euthanasia was performed. In fact, she did not even seem to understand that her life was about to be ended. In 2019, the public prosecutor charged Arends with terminating life on request and alternatively with murder. A district court acquitted her of both crimes, a decision upheld by the Supreme Court in April 2020. The geriatrician was the first physician to have been taken to court for allegedly having violated the Dutch Euthanasia Act since it came into effect in 2002.^2^

The Dutch Euthanasia Act contains a controversial clause to the effect that an advance directive can replace an actual request in case the patient has become incompetent after having drawn up a directive. The Supreme Court's verdict put a (provisional) end to the legal debate over the status of advance directives but left the broader public debate unresolved. In this article, we focus on three interrelated philosophical problems concerning the validity of advance directives: Is someone with severe dementia the same as the competent person who drew up the directive? If so, is it possible for a competent person to foresee what it will be like for her to live with dementia? And if it is, does her former self have the authority to decide about the life and death of her future self, even if that decision goes against the will of the incompetent individual she has then become? These problems are known as, respectively, the “someone else problem,”^3^ the “response‐shift problem,” and the “problem of normative authority.” But before we address them, let us first set out the most relevant facts of the case.^4^

## The Facts of the Case

In 2012, when Mrs. Cornel drew up her advance directive upon her diagnosis with early‐stage Alzheimer's, her family doctor and the geriatrician then treating her were both confident that she was competent. Three years later, she updated her request in the presence of her family doctor, who was convinced that she was still competent. Her advance directive included a standard risk‐acceptance form, containing the following clause: “By signing this euthanasia request, I fully accept the possibility that a physician acts on the request, about which I might have changed my mind had I been fully conscious.”^5^

She also signed a warrant in which she appointed her husband and daughter as her trustees, authorizing them to strive for the realization of her will as stated in her advance directive if she were no longer able to do so herself: “In that connection my trustee will bring my advance directive to the notice of my physician and see to it that she takes my request seriously and, if possible, honors it.”^6^

These clauses occur in The Dutch Right to Life Society's preprinted euthanasia form that Mrs. Cornel used for her request. That form also includes a separate dementia clause that leaves room for personal reflections concerning the circumstances in which the requester would like to have her life terminated. Mrs. Cornel's request from 2012 is handwritten; the one from 2015 is typed but still signed by her. We quote only the most relevant passages:

I want to make use of the legal right to get voluntary euthanasia when I am still passably conscious and no longer able to remain at home with my husband. I definitely do not want to be put in an institution for elderly people with dementia. I want to part from my beloved in a dignified way. My mother suffered from dementia and was treated for 12 years in an institution before she died so I have seen the process from close up. So I know what I am talking about. I definitely do not want to have this happen to me. It has seriously traumatized me and has caused the whole family great grief.^7^

The updated version from January 2015 is almost identical to the earlier one, except that the first sentence above is replaced with the following: “I want to make use of the legal right to get voluntary euthanasia when I deem the time is right.”^8^

It might seem mysterious that Mrs. Cornel included this new clause in her directive.^9^ The purpose of an advance directive, after all, can only be to serve as a substitute for your actual will when you are no longer able to express that will. At the time Mrs. Cornel drafted the directive, however, the Royal Dutch Medical Association still required that the patient confirm her euthanasia wish before the administration of the drugs, and the geriatrician present at the time had informed Mrs. Cornel of the association's policy.^10^

At the beginning of 2016, Mrs. Cornel could no longer get the necessary care at home, and in March 2016, she was admitted to a nursing home against her expressed will. Her husband notified Arends, who was employed at the nursing home, that she had an advance directive, and Arends promised to make the necessary investigations to see whether all legal due‐care requirements were met.^11^

Mrs. Cornel had spent her whole working life with children, initially as a nursery‐school teacher, later as assistant manager in a primary school. In the nursing home, she picked up her old professional habits, suggesting activities and handing out tasks to her coresidents. If they did not comply, she got frustrated and agitated, and if staff intervened, she hit, bit, and scratched them. She had always been in perfect control as a teacher and could not understand why her “children” had become so unruly. When she was upset, she had to be taken to her room, where she sat crying until staff calmed her down with great effort. Most mornings, she was tranquil, but her predominant mood was one of anxiety, anger, and despair. And she cried a lot.

Mrs. Cornel was not aware of, nor had she any insight into, her illness. She did not recognize herself in the mirror. She did, however, recognize her husband when he visited her, but she also recognized him in other visitors, in male coresidents, and occasionally even in some female ones. At night, she roamed the corridors, desperately banging on doors in search of her husband. The scenes when he left after his daily visit were heartbreaking. She was lost in the institution, could not always find the toilet, and would then defecate on the floor in other inpatients’ rooms. If she was found out, she scurried away, confused and ashamed. She could no longer dress or take care of herself and confused her mascara with toothpaste. She would tell the nursing staff that she wanted to die, on average, twenty times a day, and once suggested that it would be better if they were to hang her in a doorway, afterwards observing that it was too low. But when Arends asked her if she should help her die, Mrs. Cornel looked at her bewildered and said that this would be going too far. All in all, Arends asked her that question three times, and not once did Mrs. Cornel answer in the affirmative.

After having observed Mrs. Cornel for a month in the nursing home, Arends decided to initiate the euthanasia procedure. She consulted two independent physicians, one of whom was a psychiatrist. She also consulted the nursing‐home manager, the treatment team, and the family. All parties agreed that Mrs. Cornel was suffering unbearably, that she was incompetent, and that the conditions in her advance directive were met. The decision to comply with the directive was unanimous. Even so, Arends would not have performed the euthanasia had she not been backed up by the “Guide to Written Euthanasia Request,” published just a few months earlier by the Royal Dutch Medical Association, in cooperation with the Dutch Department of Justice and the Department of Health.^12^ The guideline expresses a view opposite that of the medical society's earlier opinion: “A patient with late‐stage dementia may be suffering unbearably from somatic complaints such as chest tightness or pain, but anxiety, aggression or agitation can also contribute to unbearable suffering. In such cases a physician can honor the euthanasia request, even if the patient is no longer able to make her request clear verbally or with gestures. It is then necessary that there exists a request written earlier by the patient.”^13^

On the morning of the euthanasia, Arends surreptitiously slipped a sedative in Mrs. Cornel's coffee to stop her from panicking or resisting. She had not told her that the euthanasia would take place that morning, nor had she sought her consent. After three‐quarters of an hour, Mrs. Cornel was still half‐awake, and Arends administered another dose subcutaneously before proceeding with a coma‐inducing drug. While she did so, Mrs. Cornel half rose, as if waking up. Her son‐in‐law gently laid her back, and Arends injected the rest of the drug. In her report to the euthanasia committee, Arends wrote that the patient resisted physically; later, in a television interview, she said Mrs. Cornel's movement could just as well be described as a shock reaction. In any case, Arends interpreted the movement as a reflex, a judgment later confirmed by an anesthetist.

## What the Court Said

Taking someone's life on that person's express and earnest request is still a crime in the Netherlands, with a maximum penalty of twelve years imprisonment, but a physician is justified in doing so if she has fulfilled all due‐care requirements stated in the Euthanasia Act of 2002. In 2019, the district court of The Hague judged that Arends had indeed fulfilled all requirements and acquitted her of all charges. In particular, the court argued that there was no legal precedent requiring that the physician should ask an incompetent requester for a confirmation of her earlier will, nor did it find such a requirement reasonable. An advance directive written by a competent person serves as a legally valid substitute for the incompetent person's actual will.^14^ The court's verdict was upheld by the Supreme Court in April 2020.

We agree that it makes no sense to ask someone so wholly incompetent as Mrs. Cornel to confirm her request. Sometimes she said she wanted to die, sometimes she said she did not, but whatever she said, she did not understand the meaning of the concept *euthanasia*, and since she did not even recognize herself in the mirror, she was certainly unable to apply the concept to herself. Nevertheless, we are in sympathy with those who find it reprehensible to kill a fully conscious human being who does not even know she is about to be killed; it smacks of murder. Our considered judgment pulls us in one direction, our intuition in another, leaving us puzzled as to what to think. Reflecting on the three questions we presented above will help us straighten things out.

## The Someone Else Problem

Is someone with severe dementia the same person as she was before she got into that state? Was Mrs. Cornel still Mrs. Cornel, or had she changed so dramatically that it would be more accurate to say that she had become someone else and that consequently the advance directive she once wrote and signed was in fact written and signed by someone who no longer existed, in which case it was not applicable to the woman she had become?

To qualify as a person, we suggest, someone must have a sufficient number of mental traits, such as autonomy, rationality, memory, and the capacity for intentional action, to a sufficient degree of complexity. If that is what it takes to be a person, what does it take to persist as one and the same person over time? What is the criterion for the numerical identity of persons? A very influential theory on personal identity, the one put forward by Derek Parfit, holds that a person at an earlier time is numerically identical to a person at a later time if and only if there is a sufficiently strong psychological connectedness between the two.^15^ Since someone in a severe state of dementia has almost no memories linking her with her earlier self, that criterion for identity is not fulfilled. So, someone with severe dementia is not the same person as she was when she drew up her advance directive.

Actually, it would stretch the meaning of the concept to apply the term “person” to someone whose mental capacities have become as impaired as had those of Mrs. Cornel, who did not even recognize herself in the mirror. Indeed, people with severe dementia are not accorded full moral status as persons; they are not held accountable for what they do, and other people withhold their reactive attitudes toward them. Others do not, for example, blame people with advanced dementia for yelling, biting, and scratching, at least not in the same way that competent individuals would be blamed (instead, with respect to such behaviors, people with severe dementia are regarded more as very young children would be). And if someone is not a person at all, she cannot, of course, be the same person as she once was.

But this does not imply that Mrs. Cornel was no longer Mrs. Cornel. Even if one acknowledges that she had changed dramatically, that she was no longer the person she used to be, perhaps not even a person at all, one still takes for granted that *she* had changed. She was still Mrs. Cornel. Physicians, caregivers, family, friends, and readers of the Dutch newspapers were all concerned about Mrs. Cornel and what had happened to *her*. Human beings are not persons all their lives. Human beings are born, but persons emerge gradually out of a pre‐person stage. And sometimes, as is the case with severe dementia, they cease to exist as persons well before they die as human beings. Persons can also cease to exist if the human being who used to be a person lives on in a permanent vegetative state or after a massive stroke. Birth and death are concepts applicable to biological entities, not to the psychological concept of a *person*.

For conceptual reasons and for practical purposes, the best criterion for numerical identity for humans is biological, not psychological. To think otherwise would have very odd implications.^16^ If humans were essentially persons, they could cease to exist before they died, and, even odder, every time that happened, a new human being would come into being. So, our answer to the questions we posed at the beginning of this section is: no, Mrs. Cornel was no longer the same person she had been, but, yes, Mrs. Cornel was still Mrs. Cornel, the same individual, and it was that individual that Mrs. Cornel had been concerned about when she drew up her advance directive. Now, do such drastic personality changes affect the authority of advance directives?

## The Problem of Response Shift

Before answering that question, however, we first want to address the problem of whether a competent person can ever foresee what it will be like to live with severe dementia. If a person were mistaken about certain relevant facts about living with dementia, her directive would consequently not be indicative of what she really wants. Recently, Emily Walsh has noted that this is particularly relevant for advance directives for people with dementia because dementia, she claims, is a “transformative experience.”^17^ She borrows that concept from Laurie Ann Paul, who has introduced it to highlight the problem of how to decide between different possible future states of affairs when those states, once they have transpired, will themselves affect how they are evaluated.^18^ Someone can ask herself, “Do I want to become a parent?,” but becoming a parent will be a transformative experience that will affect her evaluation of that new state of affairs. Since that future evaluative position is not accessible to the person now, the choice to become a parent is epistemically tricky.

People who draw up an advance directive requesting euthanasia in case they get dementia do so because they do not want to live “that way.” But, says Walsh, it is very difficult to know what it will be like to live “that way” because getting dementia constitutes a transformative experience. Although watching the Teletubbies, say, is not presently part of their idea of a life worth living, once they are afflicted with dementia, they may well enjoy it. If this is true, then there is a significant risk that their advance directive is mistaken because it is grounded on inadequate information regarding what it will be like to live with dementia. Specifically, many people believe that life with dementia will be horrible, whereas it need not be.^19^

Several points can be made here. The first concerns whether Walsh is correct in her assessment. Yes, the happily demented do exist, and Walsh is correct that people, when drawing up an advance directive, should be aware of this fact. But they should also be aware of the fact that many of those who have dementia do not enjoy their condition, and it is reasonable to consider what is most likely to occur instead of just hoping for the best.^20^ This certainly applies to Mrs. Cornel, who had seen her mother wither away in a nursing home and had two brothers who had died of Alzheimer's; it ran in her family, and she had well‐grounded apprehensions about what she could expect. Mrs. Cornel's earlier, competent self wanted to be spared her mother's horrible experiences, and her prospective judgment about her situation turned out to be correct. Everyone else involved—the geriatrician, the psychiatrist, an independent colleague, caregivers, family members, and the review committee—agreed that she was suffering unbearably.

This brings us to our second point. Is it indeed impossible to know what it will be like to undergo a transformative experience? Take Paul's example of becoming a parent. We all know people who are parents, and we can ask them about how they experience parenthood. And if they are trustworthy, they will tell us stories about the blessings and woes of being a mom or dad. And apart from what they tell us, we can observe them and make up our own minds about whether it would be worthwhile. And allowing for the necessary changes, the same holds for living with dementia. Some of us have parents or grandparents with dementia, and if they are not too severely afflicted, we can talk to them and find out how they experience their condition. If they are in a very advanced state, we can infer how they feel from their behavior. Moreover, we can read novels and plays like *Out of Mind*, *Still Alice*, and *The Father*, or watch the movies based on them. With some effort, we are able to triangulate between the facts of the matter, the mental makeup of our interlocutors, and our own attitudes reasonably successfully.^21^

Thirdly, and even more importantly, what it is like to live with dementia is not always the primary reason for drawing up an advance directive. What some people fear most is not the emotional distress or suffering, but the progressive cognitive dysfunction, memory loss, and inability to recognize even the ones they love most.^22^ Walsh's message that it might not be so bad would not console them. Even if they would later be totally contented with a much simpler life, their current self would still object to that transition. As Govert den Hartogh puts it, “For me the prospect of being happy with the Teletubbies would not detract from the horror; it would rather be its culmination.”^23^

But are people not wonderfully adaptive? The prospect of ending up in a wheelchair might seem insufferable to a lot of people, but once they find themselves in that situation, they often revise their preferences and discover that life is not as bad as they had feared it would be. They take stock and reevaluate their interests or form new ones. Is it not possible that people with dementia could also gradually reevaluate their new situation and find their lives quite sufferable or even agreeable? Actually, what follows from our previous point is that they are unable to do so. Unlike a person who ends up in a wheelchair, someone with severe dementia lacks the cognitive capacities necessary for evaluating her situation. Once afflicted, you cannot shift your response because, at “the time you would most likely ‘change your mind’, you don't have enough mind left to change.”^24^ This is what sets dementia apart from other transformative experiences and invalidates the response‐shift objection. The point is again clearly stated by den Hartogh, who uses a biblical story to illustrate the difference between dementia and other transformative experiences:

Saul's values are no longer valid for determining Paul's interests, because they have been *revoked*. But the development of dementia is not a process of conversion. If I find myself in the situation that I describe in my advance directive, I have not revoked the values expressed in that directive, they have only disappeared beyond the horizon. That is why these values are still in place for the evaluation of what my life means to me, even though I can no longer perform that evaluation myself…. The interests expressed in my living will are my categorical critical interests, and will be so at the last stage of dementia as much as they have ever been.^25^

## The Normative Authority Problem

Still, we have not addressed our final question. Even if health care professionals are dealing with the same individual (if not the same person), and even if her former self could foresee a condition she wanted to avoid and wrote an advance directive to make clear how she wanted to be treated if she lost her competence, the people caring for her must still ask what authority should be assigned to such a directive. In case of conflict, should clinicians follow the former person's directive or consider the wishes and interests of the now‐incompetent individual to be authoritative instead?

The best‐known advocate of the authority of advance directives is Ronald Dworkin.^26^ Central to his argument is the distinction between experiential and critical interests. On the one hand, he says, people have experiential interests in having pleasant rather than bad experiences. But, on the other hand, they also have critical interests in leading a meaningful life; they treasure intimate relationships, are committed to particular projects, or hold other values that they use as a standard to evaluate their lives with. These two types of interests need not come apart—a person could value the quality of her experiences and want her life to reflect this hedonistic outlook—but they *can* come apart. To maintain a friendship or to follow through on a project can be costly and painful, but friends and projects give meaning to our lives, and people will sometimes forgo a pleasure to salvage something of significance.

On Dworkin's account, in case of a conflict, critical interests should always trump experiential ones, both because the way a person wants to live tracks her critical interests and because someone else's act of prioritizing her experiential interests when they conflict with her critical ones would be an odious form of moral paternalism. Since advance directives are a person's final expression of the values she wants to see realized in her life—or, perhaps even more importantly, the evils she definitely does not want to live through—an incompetent patient's advance directive ought to be followed even if that patient turns out to be as contented as Dworkin's Margo, whom a medical student recalled as “undeniably one of the happiest people I have ever known.”^27^ Respect for autonomy and beneficence prescribe the same course of action; if everybody has the right to live according to their own values, anybody's best interests are the interests they have elevated into their critical interests. A conflict between critical and experiential interests does not generate a dilemma because they are ordered lexically. (Of course, this holds only if a person was autonomous when she drew up her directive and has not revoked it while still competent.)

Dworkin's account, however, is controversial.^28^ Some critics grant that people's critical interests normally take precedence over their experiential ones, but in the specific case of severe dementia, a conflict never comes about because experiential interests are all that is left. That claim, however, is false. It is true that someone in a sufficiently advanced stage of dementia has lost the capacity to reflect on her critical interests, to revise them, or to form new ones, but that does not mean she no longer has any critical interests. When someone becomes incompetent, her critical interests live on unless she revoked or reconsidered them while she was still competent. After all, the reason for drawing up an advance directive is to make clear, while you still can, how you want to be treated when you are no longer capable of informed decisions about such matters, and the reason for complying with such directives is that, as humans, we all have an interest in having our will respected. And precedent autonomy is in fact accepted in other cases where a person is rendered incompetent—for instance, in honoring prohibitions against life‐sustaining treatment.

But respecting precedent autonomy is sometimes easier said than done. Suppose Margo, the happiest person alive, had written an advance directive stating that if she no longer recognized her closest family members and had to be institutionalized, then she would like to have her life ended. Should her physician honor that request, promote her critical interests, and let the contented Margo die, or perhaps even kill her? Dworkin strongly suggests a positive answer, as any other course would be a violation of Margo's autonomy.^29^ Different authors have tried to mitigate this conclusion by giving some weight, in some cases, on a sliding‐scale model, to experiential interests, but for Dworkin, contravening someone's autonomous wishes remains a form of tyranny, even when done with the best intentions.

Dworkin does not distinguish between killing and letting die, but in our opinion, it is important to do so. It is not a violation of someone's autonomy not to honor her request to actively end her life. Patients do not have a right to be killed; if they did, physicians would have a duty to kill—a duty no country in the world has ever imposed on physicians. To treat someone against her will, however, is always a violation of autonomy and for that reason is a form of tyranny. The fact that someone has experiential interests does not imply that she has an interest in continuing to live, especially not if she has lost the ability to imagine herself in future situations. While alive, patients have an interest in having pleasant experiences rather than bad ones, but experiential interests do not give anyone a reason to live on, unless she has made them into her critical interests. The appeal to experiential interests as a reason for ignoring a treatment refusal marks a clear disregard for autonomy and is not clearly in the permanently incompetent patient's interests. But we are aware that people differ strongly about the weight they believe that experiential interests should be accorded, and we acknowledge that physicians confronted with a seemingly perfectly contented patient with an advance euthanasia directive face a painful dilemma.

## Supporting the Supreme Court's Decision

We began this article with a fairly detailed description to show that Arends had fulfilled the two most important criteria for lawful euthanasia in the Netherlands—that she had made sure that Mrs. Cornel's request was voluntary and well considered, and her suffering unbearable and hopeless.

We then discussed three philosophical problems associated with advance directives. The someone else problem, we argued, would be a problem only if humans were essentially persons. Human beings change in the course of a lifetime, but they do not go out of existence when their personality changes, nor does a new individual emerge at the end of such a change. What constitutes numerical identity in humans, therefore, is biology, not psychology.

Next, we discussed several aspects of the problem of response shift. First, the facts about dementia are accessible, and, second, it is not impossible to know what it would be like to live with severe dementia. Third, people who draw up an advance directive may not be primarily concerned about the experiential side of dementia. Norman Cantor and den Hartogh, even when imagining a scenario in which they would come to enjoy their future state, recoil from the prospect of living with advanced dementia. And finally, since dementia eradicates the capacities that one needs to reevaluate their critical interests or to form new ones, a response shift is no longer possible at a certain point.

As for the authority of advance euthanasia directives, we discussed and criticized two ways to defuse the possible conflict between the critical interests the person who writes such a directive wants to protect and the experiential interests she has once she is living with dementia. One way is by ordering the two kinds of interests lexically, as Dworkin does; another is by denying that patients in late‐stage dementia have any critical interests at all. But we also noted that the two kinds of interests need not conflict—and in the case we have discussed, they did not. When the conditions laid out in advance euthanasia directives are fulfilled and the patients are also suffering unbearably, the promotion of critical and experiential interests—respect for autonomy and care for the patients’ well‐being—point physicians to the same course of action Arends took.

## Acknowledgments

We wish to thank three anonymous referees for their fair and very helpful and detailed comments.
